# Removal of Lead Cations by Novel Organoclays Derived from Bentonite and Amphoteric and Nonionic Surfactants

**DOI:** 10.3390/toxics12100713

**Published:** 2024-09-30

**Authors:** Maria Gertsen, Leonid Perelomov, Anna Kharkova, Marina Burachevskaya, S. Hemalatha, Yury Atroshchenko

**Affiliations:** 1 Laboratory of Soil Chemistry and Ecology, Faculty of Natural Sciences, Tula State Lev Tolstoy Pedagogical University (Tolstoy University), Lenin Avenue, 125, 300026 Tula, Russia; perelomov@rambler.ru (L.P.); marina.0911@mail.ru (M.B.); reaktiv@tsput.ru (Y.A.); 2 Laboratory of Biogeochemistry, Faculty of Natural Sciences, Tula State Lev Tolstoy Pedagogical University (Tolstoy University), Lenin Avenue, 125, 300026 Tula, Russia; 3 Natural Sciences Institute, Tula State University, Lenin Avenue, 92, 300012 Tula, Russia; anyuta_zaytseva@mail.ru; 4 School of Life Sciences, B.S. Abdur Rahman Crescent Institute of Science and Technology, Chennai 600048, India; hemalatha.sls@crescent.education

**Keywords:** clays, surfactants, modification, adsorption, heavy metals, ecosystem remediation

## Abstract

For many decades, natural and modified clay minerals have been used as adsorbents to clean up aquatic and soil ecosystems contaminated with organic and inorganic pollutants. In this study, organoclays based on bentonite and various amphoteric and nonionic surfactants were synthesized and tested as effective sorbents for lead ions. The maximum values of R were obtained when describing the sorption processes using the Langmuir model, which ranged from 0.97 to 0.99. The adsorption of lead ions by these organoclays was investigated using different sorption models including the Langmuir, Freundlich, and BET. It was found that, according to the values of limiting adsorption to the Langmuir equation, the synthesized organoclays formed an increasing series: organoclay with cocamide diethanolamine < bentonite < organoclay with lauramine oxide < organoclay with sodium cocoiminodipropionate < organoclay with disodium cocoamphodiacetate < organoclay with alkyl polyglucoside. The Gibbs energy for all of the analyzed samples was calculated and found to be negative, indicating the spontaneity of the cation adsorption process in the forward direction. The maximum value of the adsorption capacity of lead cations on organoclay-based bentonite with alkyl polyglucoside was 1.49 ± 0.05 mmol/g according to the Langmuir model, and 0.523 ± 0.003 mmol/g as determined by the BET model. In the process of modifying bentonite, there was an increase in negative values of the zeta potential for organoclays compared to the initial mineral, which clearly enhanced their electrostatic interactions with the positively charged lead ions. It was hypothesized, based on the physicochemical principles, that exchange adsorption is the main mechanism for lead absorption. Based on chemical approaches, organoclays based on amphoteric surfactants absorb lead mainly through the mechanisms of electrostatic attraction, ion exchange, and complexation as well as the formation of insoluble precipitates. Organoclays based on nonionic surfactants, on the other hand, absorb lead through mechanisms of complexation (including chelation) and the formation of insoluble chemical precipitates. The comparison of isotherms from different models allows us to find the most accurate match between the model and the experimental data, and to better understand the nature of the processes involved.

## 1. Introduction

The widespread heavy metal pollution of environmental objects is one of the most significant environmental issues today [1]. Metals do not decompose biologically or chemically, but instead undergo transformations of their compounds, leading to their long-term presence in soil, ground, and sediment, which can become sources of secondary pollution over time. Numerous studies have demonstrated that the accumulation of heavy metals in soil can pose risks to soil fertility and quality, disrupting its ecological functions [2,3,4]. In soil environments, processes such as sorption, desorption, chemical complexation with inorganic and organic ligands, and oxidation–reduction are crucial for monitoring the availability, mobility, and toxicity of heavy metals. These reactions are influenced by various factors including pH, the composition of the soil, the presence of inorganic and organic ligands such as humic and fulvic acids, and microbial metabolites and root exudates [5,6].

Currently, a significant challenge lies in the search for and development of efficient, environmentally friendly, and cost-effective remediation techniques for soils contaminated with heavy metals. These techniques should reduce the mobility and biological activity of these pollutants in order to improve the overall health of the environment. Several approaches have been explored including the use of graphene oxide modified with chitosan to adsorb lead(II) ions [7], polymer adsorbents based on ethylenediamine, diethylenetriamine, and tetraethylenepentamine [8], carbon nanotubes [9], humic acids [10,11], biochar [12], and natural and modified clay minerals [13,14]. These materials have shown promise in reducing the concentration of heavy metals in contaminated soils. Natural and modified clay minerals with expanding structural cells have also been considered as potential remediation materials due to their ability to interact with and remove heavy metals from soil. These materials hold great potential for future research and development efforts in this area.

Natural montmorillonite has traditionally been used to absorb and immobilize various cationic pollutants such as heavy metals, dyes, and some pesticides [15]. Bentonite clay pastes and suspensions have been used to decontaminate clothing, equipment, and building materials during the clean-up of the Chernobyl disaster as well as prevent the movement of radionuclides through water [16]. It has been shown that natural clays are highly effective at absorbing Cs^+^ from radioactive waste [17].

Various physical and chemical methods for modifying clay can significantly improve its sorption properties and expand its application for new substances. Currently, there is active research underway on the sorption properties of clay minerals modified with organic substances, also known as organoclays. The sorption efficiency and strength of pollutant binding as well as the methods for synthesizing and applying organoclays are all being optimized [18,19,20].

The interaction between organic matter and clay minerals is a complex process that is influenced by the chemical nature and structural characteristics of the minerals as well as the physical and chemical properties of the organic matter. It is also influenced by the environmental conditions under which the interaction occurs. Organic matter has a significant impact on clay minerals, altering their surface through the formation of chemical bonds and intermolecular aggregates. These interactions are facilitated by hydrogen bonds and Van der Waals forces as well as physical contact. As a result, the resulting organoclay products exhibit properties that differ significantly from those of the individual starting materials. At present, the mechanisms of interaction between heavy metals and the complex organoclays have not been thoroughly investigated. The study of these mechanisms, using individual organic matter with known properties, also contributes to our understanding of the interactions between specific and nonspecific soil organic matter and the mineral components of soil in complex natural systems.

The most common way to describe adsorption processes and the properties of adsorbents and adsorbates is through the use of adsorption isotherms, which are constructed based on experimental data. These isotherms are equations that describe the equilibrium between the concentration of retained metal ions at the surface of the adsorbing material and the concentration in the surrounding solution. Equilibrium data from experiments are fitted into these equations to create adsorption isotherm models [21]. An adsorption isotherm can be used to visualize the dynamic equilibrium between these two concentrations at a given temperature and initial adsorbate concentration. It can also help to determine the type of adsorption (monolayer or multilayer) and the nature of the surface (homogenous or heterogenous). Additionally, it can distinguish between physical adsorption and chemisorption [22]. Adsorption isotherms can be used to determine the thermodynamic parameters that characterize the adsorbed layer such as the heat of adsorption, changes in entropy, and heat capacity associated with the adsorption process. In the case of physical adsorption isotherms, they can also be used to calculate the surface area of the adsorbed layer [23]. The equations for adsorption isotherms provide valuable information about the capacity of the adsorbent and the strength of the interaction between the adsorbate and adsorbent [24].

Experimental data obtained from the equilibrium distribution of the adsorbate provide specific and individual characteristics for each system. Therefore, modeling the adsorption isotherms is essential for assessing the applicability of adsorbents and designing an optimal adsorbent–adsorbate system for the desired separations [25]. Isothermal models, such as Langmuir, Freundlich, Dubinin–Radushkevich, Brunauer–Emmett–Teller (BET), Temkin, and others, are used to predict the equilibrium behavior of metal ions during adsorption on clay adsorbents [26]. Although there are many isotherm models available in the literature, it is important to select those that describe the physical and chemical properties of the adsorbing material most closely. Comparing isotherms from different models allows for a more accurate match with the experimental data and a better understanding of the underlying processes.

The aim of this study was to investigate the sorption characteristics of organomineral complexes formed by bentonite clay and amphoteric or nonionic surfactants in relation to lead ions by using adsorption isotherm models.

## 2. Materials and Methods

### 2.1. The Composition and Properties of the Initial Clays

Bentonite from the Sarigyukh deposit in Armenia (Bento Group Minerals, Moscow, Russia) was used to create the organoclays. The manufacturer states that it has the following oxide composition: SiO_2_—58.3%, Al_2_O_3_—14.3%, Fe_2_O_3_—4.4%, MgO—3.6%, Na_2_O—2.3%, K_2_O—1.2%, and CaO—2.1%. The mineral’s cation exchange capacity is 105 mg eq/100 g.

### 2.2. X-ray Diffraction Analysis

A X-ray diffractometric study of a clay mineral was conducted at the Institute of Physicochemical and Biological Problems of Soil Science of the Russian Academy of Sciences using a DRON-3 diffractometer (SPE Burevestnik, St. Petersburg, Russia). The experiment was performed under normal conditions with CuKα radiation. The survey was conducted in the angle range of 2° < 2θ < 20°, with a scanning step of 0.05°, and an exposure time of τ = 1 s. Diffractograms were taken for qualitative and semi-quantitative determinations from oriented preparations prepared by the natural orientation of clay particles on a glass substrate at normal pressure and room temperature. Three preparations with the same suspension density and thickness were studied on the substrate [27].

### 2.3. Surfactants Used for Modification

For the synthesis of organoclays based on bentonite, commercially available amphoteric and nonionic preparations of surfactants were used (ETS, Moscow, Russia) (Table 1).

These surfactants are used as detergents.

### 2.4. Organoclay Synthesis

The monoionic sodium form of bentonite was used to synthesize organoclays. To prepare the monoionic form of the mineral, 0.2 N NaCl was added to a purified natural phyllosilicate that had been crushed to particles smaller than 75 µm twice—first for 6 h, and then for another 18 h at a ratio of 1:50 (weight:volume). After the procedure was completed, the mineral was washed with deionized water several times until a negative chloride ion test with AgNO_3_ was obtained. The solid phase was separated from the liquid by centrifugation at 10,000 rpm for 10 min, and the mineral was then dried at 60 ± 2 °C until it reached a constant weight. Finally, the dried mineral was crushed into a fine powder.

A total of 1 g of the surfactant was dissolved in 95 mL of deionized water and added to 5 g of the mineral in a conical flask. The mixture was thoroughly stirred with a glass rod and then placed on a digital orbital shaker S-3M.A10 (SIA “ELMI”, Riga, Latvia) in a closed flask at a frequency of 180 strokes per minute for 24 h at a temperature of 22–23 °C. Afterward, the organoclay and the liquid phase were separated by centrifugation using a laboratory bench top centrifuge CLn-16 (Xiangzhi Centrifuge, Changsha, China) at 10,000 rpm per minute for 10 min, followed by decantation of the supernate.

The sediment was washed three times with 40 mL of deionized water in centrifuge tubes, and thoroughly stirred for 60 s with a glass rod. The liquid phase was then separated by centrifugation at 10,000 rpm for 10 min, and the supernatant was decanted. After combining the sediment in a Petri dish, the organoclay was dried at 60 ± 2 °C until it reached constant weight. This temperature was chosen to prevent the decomposition and transformation of the sorbed organic matter. After grinding the organoclay in an EM-100 laboratory mill (Vetinstrument, Rodninki, Russia), the resulting material was stored in hermetically sealed weighing bottles with a ground-in lid and placed in a desiccator.

### 2.5. Adsorption Experiment

The study of heavy metal adsorption by the synthesized organoclays was conducted through static sorption experiments. Lead cations, in the form of nitrate, were chosen as the model heavy metal. A solution of metal nitrate with varying concentrations (ranging from 0.4 mM to 4 mM) was added to 0.2 g of organoclay in a 100 mL conical flask. The experiments were performed in an electrolyte solution of 0.01 M KNO_3_ with a pH of 5 to ensure equalization of the ionic strength of the solution. The solid-to-liquid ratio was 0.2:25, and the suspension was thoroughly mixed. After mixing, the flasks were shaken on an orbital shaker at a frequency of 180 movements per minute, for 3 h, at a temperature of 22–23 ° C. After this, the organoclay and liquid phases were separated by centrifugation using a laboratory bench top centrifuge CLn-16 (Xiangzhi Centrifuge, Changsha, China) at 10,000 rpm for 10 min. The supernatant liquid was then decanted and filtered through a syringe filter with a pore size of 0.45 µm.

The content of lead ions in the liquid phase was determined using flame atomic absorption spectrometry on an Analytik Jena ContrAA^®^ 800 F spectrometer (Analytik Jena AG, Jena, Germany). The amount of metal adsorbed was calculated by comparing its concentration in the initial solution with its concentration in equilibrium. To calculate the sorption capacity of Pb^2+^ ions in the system, Equation (1) was used.

(1) $$Q=\frac{C_{0}-C}{m}\cdot V$$

where Q is the amount of absorbed cations, mmol/g; C is the equilibrium concentration of the metal with the sorbent, mmol/L; C_0_ is the analytical concentration of the metal, mmol/L; V is the volume of the solution, l; m is the weight of the adsorbent, g.

The adsorption of Pb^2+^ ions on the surface of the initial bentonite and organoclays based on amphoteric and nonionic surfactants was studied using the Langmuir, Freundlich, and BET theoretical models.

### 2.6. Determination of pH of the Salt Extract

The exchange acidity of the initial bentonite and organoclays was determined using a 1 M KCl solution at a bentonite:solution ratio of 1:5 and potentiometric measurement of the solution pH with the pH meter/ionometer ITAN (LLC NPP “Tomyaanalit”, Tomsk, Russia) [28].

### 2.7. FTIR Spectroscopy

The infrared spectra of the samples were obtained using an FSM-2202 infrared Fourier spectrometer (INFRASPEC, St. Petersburg, Russia). The spectra were collected in the range of 4000–400 cm^−1^, in transmission mode, with a resolution of 4 cm^−1^ and a number of scans of 4. The Norton–Beer average apodization technique was used. Before obtaining the spectrum for each sample, a comparison sample of pure KBr was removed [29].

### 2.8. Determination of Zeta Potential by Electrokinetic Method

Determination of the zeta potential was carried out in the Department of Fundamental Chemistry at The Novomoskovsk Institute of the D.I. Mendeleev University of Chemical Technology. The zeta potential of samples was measured using a Stabino II (Colloid metrix, Meerbusch, Germany) equipped with a microprocessor unit. The unit automatically calculates the electrophoresis mobility of the particles and converts it to the zeta potential using the Smoluchowski equation [30]. A sample of 1 g in 100 mL distilled water was added to a thermostatic shaker bath (BIOBASE, Jinan, China) and rinsed for 24 h at 20 ± 2 °C. The samples were allowed to tend for 5 min to let larger particles settle. An aliquot taken from the supernatant was used to measure the zeta potential. The average of five measurements was taken to represent the measured potential. The applied voltage during the measurement was generally varied in the range of 50–150 mV.

## 3. Results and Discussion

### 3.1. X-ray Diffraction Analysis

**Figure 1:**
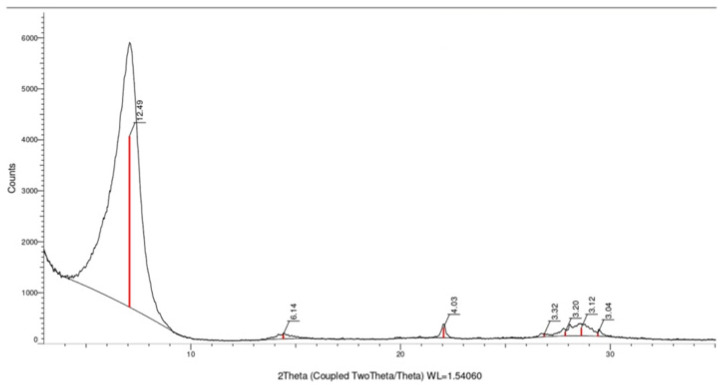
The results of the X-ray diffraction analysis of bentonite used in the synthesis of organoclays.

The results of the X-ray diffraction analysis of the bentonite used are shown in Figure 1. The analytical data indicate a high content of montmorillonite (12.49 Å) and minor impurities of quartz (3.32 Å) and feldspar (3.20 Å).

When the initial bentonite is converted to the monoionic sodium form, the distance between particles remains practically unchanged (d001 = 12.45 Å). Therefore, the physical distance between particles with a silicate plate thickness of 9.6 Å in the monoionic form is approximately 2.85 Å. 

### 3.2. IR Spectroscopy

**Figure 2:**
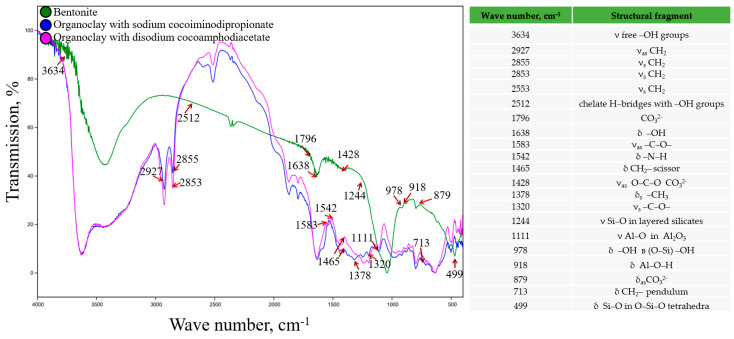
IR spectra of the synthesized organoclays using amphoteric surfactants.

According to the IR spectroscopy data (Figure 2), several characteristic absorption bands were observed in the spectrum of the initial bentonite. These included bands at 978 and 1197 cm^−1^, which corresponded to free and associated forms of the Si–OH groups. The absorption band at 1638 cm^−1^ was related to the stretching and deformation vibrations of the OH group. The bands in the region of 798–838 cm^−1^ were caused by the vibrations of the Si-O bond in tetrahedral molecules, while the bands in the range 426–439 cm^−1^ corresponded to the vibrations of this bond within the tetrahedron [31]. The bands between 3000 and 3700 cm^−1^ represented the stretching and deformation of OH groups in free or bound water molecules, while the absorption at 2512 cm^−1^ corresponded to the formation of chelate H-bridges with OH groups. Additionally, characteristic absorption bands for Al-O groups in Al_2_O_3_ (1111 cm^−1^) and Si-O-Al stretches (645–669 cm^−1^) were also observed as well as those related to O-C-O vibrations in CO_3_^2−^ ions (1197 cm^−1^) [32].

The IR spectra of bentonite samples treated with amphoteric surfactants showed absorption bands that were characteristic of the original mineral form. However, there was a decrease in the number of free -OH groups, which was likely due to the distribution of surfactant molecules within the interlayer spaces of bentonite and the displacement of water that was previously located there. The absorption bands at 1465, 2853–2927, and 1378 cm^−1^ corresponded to vibrations of the CH_2_ bond and CH_3_, respectively, indicating the presence of organic compounds within the structure. Additionally, the appearance of absorption bands at 1542, 1320, and 1583 cm^−1^ was related to amino groups and carboxylate acid residues (Figure 2).

**Figure 3:**
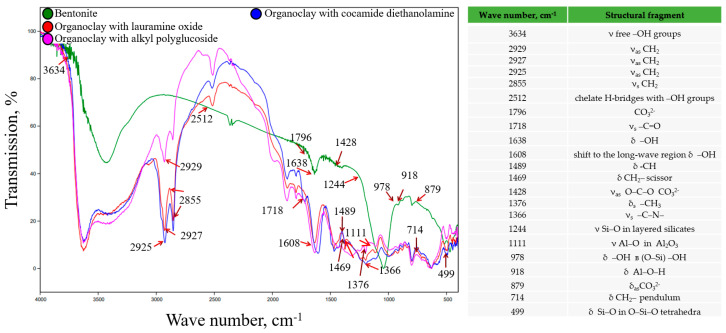
IR spectra of the synthesized organoclays using nonionic surfactants.

For organoclays synthesized with the participation of nonionic surfactants, a similar situation was observed as with the modification by amphoteric surfactants (Figure 3).

In addition, there was a shift to the long-wave region of hydroxyls (1608 cm^−1^), carbonyls (-C=O 1718 cm^−1^), and absorption bands 2855–2929 cm^−1^, belonging to asymmetric and symmetric stretching vibrations of the CH_2−_ group of the adsorbed surfactant.

### 3.3. The Acid–Base Properties of the Original and Modified Bentonite

When characterizing the cation exchange capacity of bentonite and the organoclays, the acid–base properties of the samples were determined. The most significant factor for the cation exchange process is the exchange acidity, which is determined in a salt extract. H^+^ and Al^3+^ ions from this type of acidity are the first to enter into exchange reactions and compete with exchange cations. The results of the acid–base property determination are presented in Table 2.

The pH values of the aqueous and salt suspensions of the original and modified forms of bentonite were alkaline, ranging from 8.19 ± 0.02 to 10.92 ± 0.04. The original form of bentonite had a slightly alkaline environment (aqueous pH of 8.38 ± 0.04). The salt extract had a pH of 9.78 ± 0.01, indicating the presence of some exchange acidity due to the presence of mobile exchange ions in the material.

The modification of bentonite with different types of surfactants (amphoteric and nonionic) affected the dehydration of the surface functional groups of the bentonite. The pH of the solutions of the aqueous and salt extracts from the modified samples shifted toward the alkaline region to varying degrees

### 3.4. Isotherm Studies

An accurate representation of the dynamic adsorption process of a solute on an adsorbent depends on the description of its equilibrium distribution between the two phases. Equilibrium is reached when the amount of solute adsorbed on the adsorbent is equal to the amount disturbed in the solution. The equilibrium concentration of the solution remains constant. The graph displaying the dependence of the concentration of the adsorbed solute in the solid phase and the concentration in the liquid phase shows the equilibrium adsorption isotherm [33,34,35,36].

Sorption equilibrium isotherms are an important tool for analyzing and designing adsorption systems and provide a way to assess and interpret thermodynamic parameters. While isotherms may accurately match the experimental data under certain conditions, they can completely disagree under other conditions. This paper presents the results of fitting adsorption isotherms using the Langmuir, Freundlich, and BET models as they allowed us to find the best possible fit between the experimental and theoretical data.

#### 3.4.1. The Langmuir Isotherm

The Langmuir adsorption isotherm theory assumes that a monolayer of the adsorbate covers a uniform surface of the adsorbent. This is represented graphically by a plateau in the isotherm. The model states that at equilibrium, the adsorption reaches a saturation point where no further absorption can occur. Equation (2) was used to analyze the experimental data on adsorption processes within the context of the Langmuir model.

(2) $$\;Q=Q_{\infty }\;\frac{\;K_{L}C_{eq}}{\left(1+K_{L}C_{eq}\right)}$$

where Q is the amount of absorbed cations, mg/g; Q_∞_ is the maximum adsorption value of an element, mmol/g; K_L_ is the Langmuir constant, L/mmol; C_eq_ is the concentration of an element in an equilibrium solution, mmol/L.

**Figure 4:**
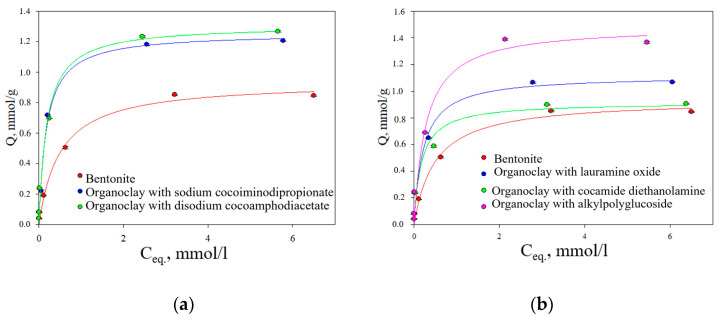
Adsorption isotherms of Pb^2+^ ions on the initial and surfactant-modified bentonite forms: (**a**) organoclays modified with amphoteric surfactants; (**b**) organoclays modified with nonionic surfactants.

The obtained sorption isotherms are shown in Figure 4.

The Langmuir adsorption isotherms for Pb^2+^ ions on bentonite and its modified forms with the participation of amphoteric and nonionic surfactants followed the L-type according to the Giles classification [37]. The shape of the isotherm at low concentrations indicates that the binding strength to the sorbent is relatively strong. At the beginning of the process, there is an excess of available functional groups for binding and a high concentration of metal in the solution. As equilibrium is approached, functional groups become occupied by metal ions, and filling the adsorption surface becomes more difficult due to repulsive forces between the bound metal ions and those still present in solution [38]. The parameters of the Langmuir isotherm can be found in Table 3.

It was found that high correlation coefficients (above 0.97) indicated the applicability of the Langmuir model for describing the adsorption of lead ions on the surface of the initial bentonite and organoclays. Based on the value of maximum adsorption (A_∞_), the sorbents studied with the use of amphoteric surfactants can be arranged in the following order: bentonite, organoclay containing sodium cocoamphodiacetate, and organoclay containing disodium cocoamphodiacetate. The parameter K_L_, which describes the strength of the bond between metal ions and functional centers on the sorptive surface, increased in the series: bentonite < organoclay with disodium cocoamphodiacetate < organoclay with sodium cocoiminodipropionate.

In the case of modification with nonionic surfactants, the sorbents analyzed can be arranged in order of increasing A_∞_ value as follows: organoclay with cocamide diethanolamine < bentonite < organoclay with lauramine oxide < organoclay with alkyl polyglucoside. Although the maximum calculated adsorption capacity of the organomineral complex involving diethanolamine cocamide may have been lower than that of bentonite, it was still absorbed quite effectively by this organoclay at relatively low concentrations of metal (Figure 4b). Based on the K_L_ value, the order is: bentonite < organoclay with alkyl polyglycoside < organoclay with lauramine oxide < organoclay with cocamide diethanolamine.

In terms of maximum adsorption, organoclays synthesized using both amphoteric and nonionic surfactants formed a series in ascending order: organoclay with cocamide diethanolamine (nonionic surfactant) < bentonite < organoclay with lauramine oxide (nonionic surfactant) < organoclay with sodium cocoiminodipropionate (amphoteric surfactant) < organoclay with disodium cocoamphodiacetate (amphoteric surfactant) < organoclay with alkyl polyglucosides (nonionic surfactant).

The Langmuir constant varied in the following order for both types of surfactants: bentonite < organoclay with alkyl polyglucoside (nonionic surfactant) < organoclay with lauramine oxide (nonionic surfactant) < organoclay with disodium cocoamphodiacetate (amphoteric surfactant) < organoclay with sodium cocoiminodipropionate (amphoteric surfactant) < organoclay with diethanolamine cocamide (nonionic surfactant). Therefore, based on the K_L_ value, both amphoteric and nonionic surfactants improved the bond strength between bluestone and the organometallic complex when compared to the original bentonite.

The data obtained on the adsorption of Pb^2+^ ions by the initial form of bentonite and the synthesized organoclays from aqueous solutions can be compared with the literature data for existing analogs (Table 4):

Comparison of the obtained data with analogous materials (within the studied range of concentrations and pH) led to the conclusion that the synthesized organoclay materials could be used as efficient sorbents for Pb^2+^ ions.

#### 3.4.2. The Freundlich Isotherm

The Freundlich model is used to describe adsorption behavior in heterogeneous systems. The surface of natural sorbents is believed to be non-uniform, with several different sorption sites characterized by different energy levels [42]. The main limitation of the Freundlich model lies in the fact that the adsorption capacity is not fully saturated, leading to a continuous increase in adsorption. Consequently, the parameters of the Freundlich equation can only provide information about the degree of surface non-uniformity, and indirectly, the number of adsorption sites [43].

The two-parameter Freundlich sorption model is described by the exponential Equation (3):

(3) $$Q=K_{F}C_{eq}^{1/n}$$

where Q is the amount of absorbed cations, mmol/g; K_F_ is the Freundlich constant, l/mmol; C_eq_ is the concentration of an element in an equilibrium solution, mmol/L; 1/n is the empirical exponent.

This expression is characterized by a heterogeneity factor of 1/n, which allows the Freundlich isotherm to be used to describe heterogeneous systems. Theoretically, an infinite amount of adsorption can occur using this expression [34].

**Figure 5:**
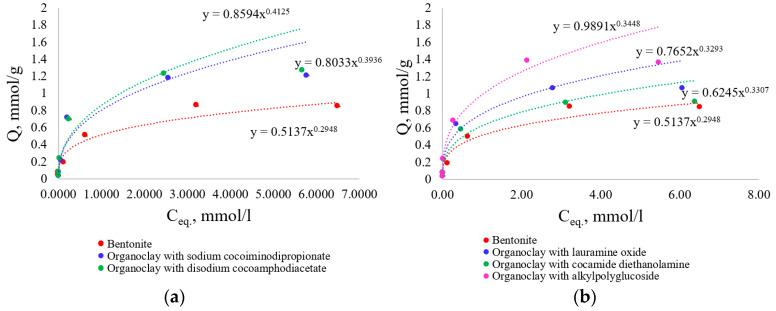
Adsorption isotherms of Pb^2+^ ions on the initial and surfactant-modified forms of bentonite: (**a**) organoclays modified with amphoteric surfactants; (**b**) organoclays modified with nonionic surfactants.

The Freundlich isotherms and parameters for this model are presented in Figure 5 and Table 5.

Based on the correlation coefficients, the Freundlich model is less suitable for describing lead ion adsorption processes on organoclay surfaces than the Langmuir model. The coefficient K_F_, which measures absorption capacity, increased in the following order when the organoclays were modified with amphoteric surfactants: bentonite < organoclay with sodium cocoiminodipropionate < organoclay with disodium cocoamphodiacetate. In the case of nonionic surfactant modification, the sorbents analyzed can be arranged in order of increasing K_F_ value as follows: bentonite < organoclay with cocamide diethanolamine < organoclay with lauramine oxide < organoclay with alkyl polyglucoside. The dimensionless parameter 1/n can be used to identify the energy heterogeneity of reaction centers on sorption surfaces, and its value can vary between 0 and 1. When 1/n is close to 0, there is high heterogeneity among the sorption sites, while when it is closer to 1, the sites become more homogeneous. The specified parameter for all analyzed sorbents varied within the range from 0.295 ± 0.003 to 0.413 ± 0.005, indicating an increase in the heterogeneity of the sorption layer.

The high correlation coefficients and 1/n values indicate that the Pb^2+^ ions were more effectively adsorbed onto the organoclays compared to the original bentonite form. This was likely due to a redistribution of electron density within the adsorbent atoms, leading to the formation of active adsorption sites on the surface.

#### 3.4.3. The BET Isotherm

Both the Langmuir and Freundlich models have drawbacks: the data on sorption equilibrium at different concentrations cannot be accurately described by a single set of parameters. The Langmuir model allows us to calculate the limiting adsorption capacity, which is useful in systems where all adsorption sites have the same energy level and ability to bind heavy metals. However, the Freundlich model is better suited for describing processes occurring on heterogeneous surfaces with multilayer adsorption. It cannot calculate the maximum adsorption capacity, but it can provide information about the heterogeneity of the surface. The BET model addresses the limitations of both models by considering energy heterogeneity on porous surfaces. Adsorbed molecules often interact with each other, leading to a violation of stoichiometry and limiting adsorption to the formation of multiple layers. The molecules of the first layer adhere to the surface of the adsorbent due to the intermolecular interaction between the adsorbent and adsorbate. Each adsorbed molecule in the first adsorption layer can act as a center for adsorption of molecules in the second layer, and so on. This process leads to the formation of subsequent sorption layers, for which physical sorption is possible. Therefore, the value of A_∞_ becomes more accurate compared to the Langmuir model.

To describe polymolecular adsorption, the Brunauer–Emmett–Teller (BET) theory was used. The isotherm equation can be expressed as follows:

(4) $$Q=\frac{Q_{\infty }\cdot c\cdot (C/C_{0})}{\left(1-C/C_{0}\right)\cdot [1+\left(c-1\right)\left(C/C_{0}\right)]}$$

where Q is the adsorption value, mmol/g; Q_∞_ is the maximum adsorption capacity of monolayer, mmol/g; C is a constant for a given adsorption system, directly related to the heat and entropy of adsorption; C, C_0_ is the equilibrium and initial concentration of lead (Pb^2+^) ions, mmol/L.

The BET adsorption equation in linear form is shown in Equation (5):

(5) $$\frac{C/C_{0}}{Q(1-C/C_{0})}=\frac{1}{Q_{\infty }\cdot c}+\frac{c-1}{Q_{\infty }\cdot c}\cdot C/C_{0}$$

**Figure 6:**
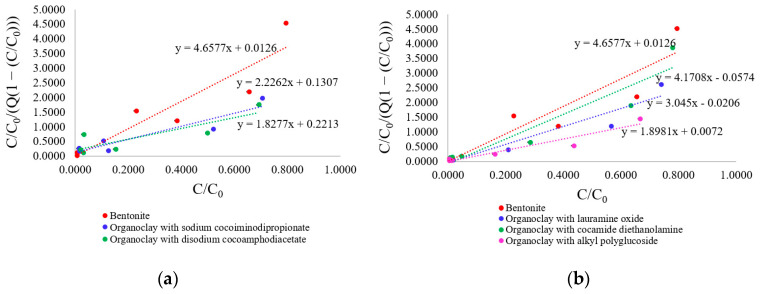
Adsorption isotherms of Pb^2+^ ions on the original and surfactant-modified forms of bentonite: (**a**) organoclays modified with amphoteric surfactants; (**b**) organoclays modified with nonionic surfactants.

The results of the experiment on the adsorption of lead ions onto clay particles were analyzed using the BET equation, which was applied in a linear form (Figure 6).

From the linear dependencies shown in Figure 6, the values of the maximum adsorption (maximum adsorption capacity of the monolayer) (A_∞_) and the constant (c) were determined. This was conducted by using the tangent of the angle of inclination of the straight lines obtained, and the size of the segments cut off on the y-axis (Table 6).

It has been established that the correlation coefficients for describing the sorption process using the BET equation are lower than those obtained using the Freundlich and Langmuir models. This is due to the fact that the BET model is only applicable in the region of high concentrations, and therefore is not suitable for describing sorption processes at low concentrations. Based on the value of the maximum adsorption (A_∞_), the order of the studied sorbent materials can be arranged as follows: bentonite < organoclay with cocamide diethanolamine < organoclay with lauramine oxide < organoclay with sodium cocoiminodipropionate < organoclay with disodium cocoamphodiacetate < organoclay with alkyl polyglucoside. The constant of the BET equation—c, which characterizes the ratio of the energy of intermolecular interactions between the sorbent (initial and modified clays) and Pb^2+^ ions, increased in the following order: organoclay with lauramine oxide < organoclay with cocamide diethanolamine < organoclay with sodium cocoiminodipropionate < organoclay with disodium cocoamphodiacetate < organoclay with alkyl polyglucoside < bentonite. Additionally, for organoclays derived from lauramine oxide and cocamide diethanolamine, the values of c were less than 1, at −0.146 ± 0.004 and −0.071 ± 0.001, respectively. When c is less than 1, even before the formation of a monolayer of adsorbed molecules is complete, the formation of a polymolecular layer begins.

Since the sorption process is best described by the Langmuir equation (with the highest coefficients of determination), the adsorption equilibrium constants obtained were used to calculate the standard Gibbs free energy (Formula (6), Table 7).

ΔG = −RTlnK(6)

where ΔG is the Gibbs energy, kJ/mol; R is the universal gas constant, J/(mol K); T is the temperature, 298 K, kJ/mol, K is the Langmuir constant.

The results obtained indicate that the adsorption of Pb^2+^ ions on both the original bentonite form and on the synthesized organoclays occurred spontaneously in the forward direction.

In our case, we observed the highest correlation coefficients when using the Langmuir model to describe the adsorption processes. This model assumes monomolecular adsorption, heterogeneity of the adsorbent surface, and predominant interaction due to chemical forces. A number of studies have also shown that the adsorption of Cu(II) onto organoclays with zwitterionic surfactants is better described by the Langmuir model compared to the Freundlich model for adsorption isotherms [44]. At the same time, [45] suggested that the adsorption of Pb^2+^ onto montmorillonite modified with different chain length betaines could be well-described by the Freundlich adsorption isotherm. The Langmuir and Freundlich R^2^ calculated from Cu^2+^, Pb^2+^, Ni^2+^, Cd^2+^, Fe^2+^, and Zn^2+^ isotherms at the metal adsorption on natural zeolite (clinoptilolite) modified by nonionic surfactant Triton X-100 showed that for Pb, Cd, and Fe, the adsorption data were fitted equally well by both the Langmuir equation and the Freundlich equation. Adsorption data for Cu, Ni, and Zn were fitted better by the Freundlich adsorption isotherm [46].

### 3.5. Determination of the Zeta Potential of Bentonite and Organoclays

The electrokinetic or zeta potential (ζ potential) is the voltage on the slip plane, which is the difference in charge between the bulk fluid and the fluid layer containing ions that are attached to the surface of a particle. For molecules and nanoparticles, a higher ζ potential means greater stability, as the solution or suspension will be less likely to aggregate. Conversely, when the ζ potential is lower, the attraction between particles exceeds the repulsive force, disrupting the stability of the suspension. Therefore, colloids with high ζ potentials are electrically stable, while those with low ζ potentials tend to coagulate or form flocs [47]. For stability, it does not matter whether the variance has a positive or negative sign; however, the sign of the variance can have a huge impact on the application of the variance.

It is now recognized that a zeta potential of at least 30 mV (optimum > 60 mV) is required for complete electrostatic stabilization, while potentials between 5 and 15 mV are in the region of limited flocculation, and those between 5 and 3 mV correspond to maximum flocculation [48]. It is important to note that the magnitude of the charge on the surface of the nanoparticles depends on the pH of the solution.

Table 8 presents the change in the zeta potential of the original bentonite and its modified versions, which were obtained using amphoteric and nonionic surfactants.

The measured zeta potential of the initial bentonite sample was −37 ± 1 mV, indicating resistance to aggregation of its negatively charged particles. When the bentonite was modified with amphoteric and nonionic surfactants, the negative values of the zeta potential increased, ranging from −49 ± 2 to −73 ± 1 mV. This indicates an increase in the stability of the bentonite suspension and a greater possibility of electrostatic interaction between the negatively charged bentonite particles and positively charged lead ions.

### 3.6. Possible Mechanisms of Lead Cation Sorption on Synthesized Organoclays

Modern concepts of the structure of the interface between solid mineral particles and the solution as well as the mechanisms of interaction between the solid and liquid phases are based on two main methodological approaches [49,50]. The first of these uses the principles and concepts of colloid chemistry, which underlie the theory of the electrical double layer [51]. The second approach is based on the theoretical principles of coordination compound chemistry. When using this approach, the reactions of interaction between solid soil particles and solution components are considered as processes of the formation of surface complexes [52].

According to the first approach, the more efficient absorption of lead cations by the colloidal particles of organoclays compared to the colloidal particles of the original clay mineral can be explained by the increase in the negative charge of the colloidal particles. The zeta potential, which is not equal to the adsorption potential or surface potential in the double electric layer, is often used to assess the properties of this layer. An increase in the zeta potential indicates an increase in colloid charge, which is associated with specific adsorption. In this type of adsorption, ions are adsorbed on the surface of the solid phase without releasing an equivalent number of ions into the solution, leading to the acquisition of an electric charge by the solid phase. This leads to the fact that, near the surface, due to the action of electrostatic forces of attraction, an equal number of ions with opposite charges are grouped, forming a double electric layer.

According to the Paneth–Fajans–Hahn adsorption rule, ions that are capable of completing their crystal lattice or forming a poorly soluble compound with one of the ions present in the crystal are specifically adsorbed onto the surface of a crystalline solid from an electrolyte solution. Lead is unlikely to be incorporated into the crystal structure of modified bentonite, but the formation of precipitates of lead hydroxides or salts is possible (see below). The observed acidification of the equilibrium solution during lead adsorption, compared to the organoclay solution, may indicate a predominant exchange nature of metal adsorption by organoclays. In general, the addition of salts containing higher-valence cations (less hydrated) can displace hydrogen and other metal ions from the surface of the micelles [53].

In bentonite, the permanent negative charge is quite high, accounting for 90–95% of the total charge. This high negative charge density is due to a significant degree of isomorphic substitution, where Fe^2+^ or Mg^2+^ replace the Al^3+^ element in the octahedral layer, and some Al^3+^ ions replace Si^4+^ ions in the tetrahedral layer. These permanent charges are mainly located on the basal surface and account for most of the surface charge density of bentonite. Positive charges are mainly located at the edges of the clay structures and only account for 5–10% of the total charge [54]. According to the second approach, positively charged fragments of amphoteric surfactants interact with negatively charged groups on the surface of bentonite, while their own anionic fragments remain unoccupied and can interact with lead cations. In this scenario, each molecule of the amphoteric surfactant contains two carboxyl groups.

During the synthesis of organoclays, surfactant molecules are added and adsorbed on the clay surface as well as penetrate (at least partially) into the interlayer space. The authors in [54] investigated the effect of the length of the carbon chain in the amphoteric surfactant, betaine, on the interlayer spacing of clays. They found that the increase in basal spacing was most significant when betaine molecules were first introduced into the galleries, but then changed only slightly as the carbon chain length increased. Therefore, an increase in betaine molecule size forced them to reorganize within the existing space rather than expanding the galleries further. The adsorption of amphoteric surfactants into interlayer spaces occurs in a monolayer, and it should be noted that we refer to small carbon chains within the molecule in this case.

The authors in [55], based on the results of infrared (IR) and X-ray photoelectron spectroscopy (XPS) studies and sorption experiments, proposed several mechanisms of interaction between Cd^2+^ and bentonite or organoclay including electrostatic interactions, ion exchange, surface complexation, and chelation. Chelation is also responsible for the adsorption of Cd^2+^ onto organoclays that contain amphoteric surfactants.

Theoretically, electrostatic interactions between molecules of nonionic surfactants and clay minerals should be absent. However, the synthesis of organoclays using nonionic surfactants is a widely used process [20,56,57]. Compared to ionic surfactants, clays modified with nonionic compounds have a hydrophobic surface without changing the degree of surface charge [56]. Organoclays synthesized with nonionic surfactants are a type of organomineral complex whose formation is not based on ion exchange. Instead, the authors suggest that the nonionic surfactant molecules interact with the silicate through their functional groups and with water molecules associated with exchange cations in clay minerals through ion-dipole and hydrogen bonds [58]. It was also noted that nonionic surfactants adsorb onto clay surfaces due to the combination of polar attractions, Van der Waals forces, and weak C-H…O (clay) bonds [59]. This can lead to the displacement of water molecules and the formation of organic layers within the interlayer space. X-ray diffraction analysis of systems containing nonionic surfactants and bentonite has shown that these surfactants can cause a slight increase in the spacing between the layers of smectite [58].

**Figure 7:**
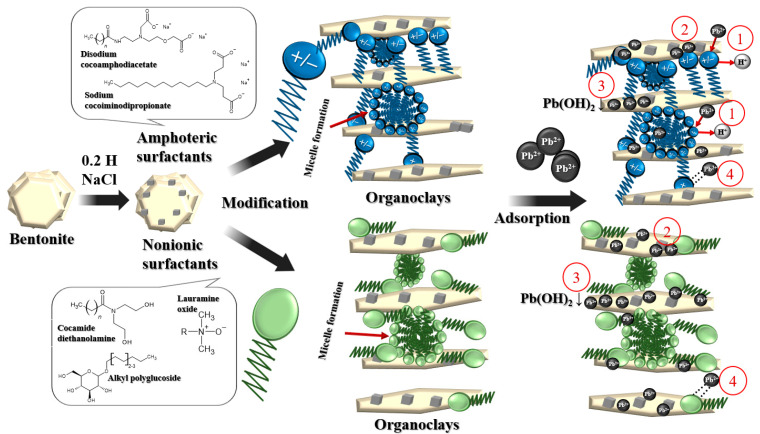
Schematic illustration of the possible mechanism for the adsorption of Pb^2+^ onto organoclays modified with amphoteric and nonionic surfactants: 1—ion exchange; 2—complexation; 3—precipitation; 4—long-range forces (physical adsorption).

In our opinion, the main mechanism of interaction between organoclays based on nonionic surfactants and lead cations involves the formation of complex compounds including chelate complexes between the structures of surfactant molecules adsorbed on the clay and the cations. Hypothetical modeling of Pb^2+^ adsorption onto synthesized organoclays can be represented as follows (Figure 7).

During the sorption experiment, a decrease in the pH of the equilibrium solutions was observed as a result of the interaction between the initial working solutions of lead nitrate and the mineral or organic clays based on both amphoteric and nonionic surfactants. The pH of the solutions decreased from 8.51 ± 0.05 to 5.11 ± 0.08, depending on the type of surfactant used for modification. This was compared to the pH values of the salt extracts, which are shown in Table 2. It is known that at pH > 6, it is possible for lead cations to form the Pb(OH)^+^ complex or the Pb(OH)_2_ precipitate, as shown in Figure 7(2) and Figure 7(3), respectively [60,61]. The XRD patterns also indicated the formation of lead carbonate hydroxide (hydrocerussite) as a result of Pb adsorption on Na-bentonite under similar experimental conditions [62].

## 4. Conclusions

The structural sorption properties of organoclays based on bentonite and various amphoteric (sodium cocoiminodipropionate, disodium cocoamphodiacetate) and nonionic (lauramine oxide, cocamide diethanolamine, alkyl polyglucoside) surfactants with respect to lead ions were synthesized and investigated. According to the Fourier transform infrared spectroscopy (FTIR) data, it was found that when bentonite is modified with these amphoteric and nonionic surfactants, there is no replacement of the existing absorption bands in the original bentonite structure.

A decrease in absorption in the long-wave region of hydroxyl groups was observed due to the distribution of surfactant molecules in the interlayer space of bentonite and the displacement of water located there.

The sorption properties of bentonite and the synthesized organoclays were investigated using various sorption models such as the Langmuir, Freundlich, and BET. It was found that the maximum adsorption capacity for lead cations in organoclays with different surfactants was observed, with the highest value being 1.49 ± 0.05 mmol/g according to the Langmuir model, and 0.523 ± 0.003 mmol/g according to the BET. The Langmuir constants, which characterize the strength of the binding between lead ions and the organoclays, varied in the following order: bentonite < organoclay with alkyl polyglucoside < organoclay with lauramine oxide < organoclay with disodium cocoamphodiacetate < organoclay with sodium cocoiminodipropionate < organoclay with cocamide diethanolamine. For the analyzed sorbents, the value of the 1/n parameter (the Freundlich equation) varied between 0.295 ± 0.003 and 0.413 ± 0.005, which indicates an increase in the heterogeneity of the sorption layer.

The negative values of the zeta potential of the synthesized organoclays increased in modulus compared to the initial bentonite (−49 ± 2 to −73 ± 1 mV) and were related to the limiting adsorption values, indicating an increase in suspension stability and an increase in the possibility of electrostatic interaction between organoclays and positively charged lead ions.

Based on the zeta potential values, structural formulas used for the synthesis of organoclays and the literature data, we made assumptions about the possible mechanisms for increasing the maximum adsorption of lead by organoclays based on amphoteric and nonionic surfactants.

## Figures and Tables

**Table 1:** Surfactants used for the synthesis of organoclays.

Name	Formula
**Amphoteric surfactants**	
Disodium cocoamphodiacetate	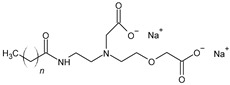
Sodium cocoiminodipropionate	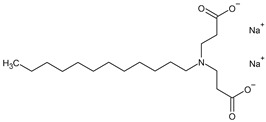
**Nonionic surfactants**	
Lauramine oxide	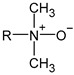
Cocamide diethanolamine	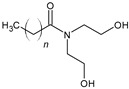
Alkyl polyglucoside	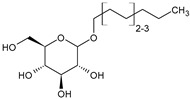

**Table 2:** The acid–base properties of the original and modified bentonite.

Samples	pH of the Aqueous Extract	pH of the Salt Extract
Bentonite	8.37 ± 0.05	9.79 ± 0.05
Organoclay with sodium cocoiminodipropionate	8.38 ± 0.04	9.78 ± 0.02
Organoclay with lauramine oxide	8.19 ± 0.02	9.81 ± 0.02
Organoclay with cocamide diethanolamine	8.62 ± 0.05	10.92 ± 0.04
Organoclay with disodium cocoamphodiacetate	8.41 ± 0.02	9.78 ± 0.01
Organoclay with alkyl polyglucoside	8.63 ± 0.01	10.10 ± 0.01

**Table 3:** Sorption process parameters based on the Langmuir equation.

Sorbent	R	Q_∞_, mmol/g	K_L_, L/mmol
Bentonite	0.99	0.95 ± 0.03	2.03 ± 0.04
With the use of amphoteric nonionic surfactants			
Organoclay with sodium cocoiminodipropionate	0.99	1.26 ± 0.07	5.67 ± 0.01
Organoclay with disodium cocoamphodiacetate	0.99	1.31 ± 0.04	5.41 ± 0.09
With the use of nonionic surfactants			
Organoclay with lauramine oxide	0.97	1.12 ± 0.04	4.67 ± 0.02
Organoclay with cocamide diethanolamine	0.98	0.92 ± 0.04	5.81 ± 0.06
Organoclay with alkyl polyglucoside	0.98	1.49 ± 0.05	3.61 ± 0.05

**Table 4:** Comparison of the adsorption capacity of the synthesized organoclays for Pb^2+^ ions with the best sorption capacities of different adsorbents.

Sorbent	Q_∞_, mg/g
Bentonite (this work)	194.5
Bentonite (Saudi Arabia)	51.19 [39]
Magnetically modified zeolite	123 [40]
Green AC/HKUST-1 Nanocomposite	249.4 [41]
Organoclay with disodium cocoamphodiacetate	271.2
Organoclay with alkyl polyglucoside	380.8

**Table 5:** Sorption process parameters based on the Freundlich equation.

Sorbent	R	K_F_, L/mmol	1/n
Bentonite	0.99	0.514 ± 0.004	0.295 ± 0.003
With the use of amphoteric nonionic surfactants			
Organoclay with sodium cocoiminodipropionate	0.97	0.803 ± 0.006	0.394 ± 0.003
Organoclay with disodium cocoamphodiacetate	0.97	0.859 ± 0.008	0.413 ± 0.005
With the use of nonionic surfactants			
Organoclay with lauramine oxide	0.98	0.765 ± 0.003	0.329 ± 0.002
Organoclay with cocamide diethanolamine	0.98	0.625 ± 0.008	0.331 ± 0.004
Organoclay with alkyl polyglucoside	0.98	0.989 ± 0.004	0.345 ± 0.003

**Table 6:** Sorption parameters of the process according to the BET equation.

Sorbent	R	Q_∞_, mmol/g	c·10^−3^
Bentonite	0.96	0.214 ± 0.002	0.37 ± 0.01
With the use of amphoteric surfactants			
Organoclay with sodium cocoiminodipropionate	0.96	0.422 ± 0.004	0.018 ± 0.002
Organoclay with disodium cocoamphodiacetate	0.93	0.491 ± 0.003	0.091 ± 0.002
With the use of nonionic surfactants			
Organoclay with lauramine oxide	0.97	0.328 ± 0.002	−0.146 ± 0.004
Organoclay with cocamide diethanolamine	0.97	0.241 ± 0.002	−0.071 ± 0.001
Organoclay with alkyl polyglucoside	0.98	0.523 ± 0.003	0.264 ± 0.006

**Table 7:** Gibbs energy (Langmuir model).

Sorbent	ΔG^0^, kJ/mol
Bentonite	−1.7
Organoclay with sodium cocoiminodipropionate	−4.2
Organoclay with disodium cocoamphodiacetate	−4.1
Organoclay with lauramine oxide	−3.8
Organoclay with cocamide diethanolamine	−4.3
Organoclay with alkyl polyglucoside	−3.1

**Table 8:** The zeta potential of the original bentonite and the synthesized organoclays.

Sorbent	ζ, mV
Bentonite	−37 ± 1
With the use of amphoteric surfactants	
Organoclay with sodium cocoiminodipropionate	−68 ± 2
Organoclay with disodium cocoamphodiacetate	−73 ± 1
With the use of nonionic surfactants	
Organoclay with lauramine oxide	−49 ± 2
Organoclay with cocamide diethanolamine	−64 ± 2
Organoclay with alkyl polyglucoside	−70 ± 3

## Data Availability

All data are included in the manuscript.
